# Supplementary material for: Origami metamaterials for ultra-wideband and large-depth reflection modulation
Source: Nat Commun. 2024 Apr 12;15:3181. doi: 10.1038/s41467-024-46907-3 (PMC11015009; doi:10.1038/s41467-024-46907-3)
Supplement: Supplementary file 1 — Supplementary information [file 41467_2024_46907_MOESM1_ESM.pdf]

Origami Metamaterials for Ultra-Wideband and Large-Depth Reflection Modulation

Zicheng Song[1,2,#], Juan-Feng Zhu[3,#], Xianchao Wang[4,#], Ruicong Zhang[1,2], Pingping Min[1,2], Wenxin Cao[1,2], Yurong He[5], Jiecai Han[1], Tianyu Wang[5]*, Jiaqi Zhu[1,2]*, Lin Wu[3,7]* and Cheng-Wei Qiu[6,]*

[1] *Center for Composite Materials and Structures, Harbin Institute of Technology, Harbin 150080, China*
[2] *Zhengzhou Research Institute, Harbin Institute of Technology, Zhengzhou 450018, China*
[3] *Science, Mathematics, and Technology Singapore University of Technology and Design (SUTD) 8 Somapah Rd, 487372 Singapore*
[4] *School of Mathematics, Harbin Institute of Technology, Harbin 150080, China*
[5] *School of Energy Science & Engineering, Harbin Institute of Technology, Harbin 150080, China*
[6] *Department of Electrical and Computer Engineering, Faculty of Engineering National University of Singapore 117583 Singapore*
[7] *Institute of High Performance Computing (IHPC) 138632 Singapore*
[#] *These authors contributed equally: Zicheng Song, Juan-Feng Zhu, Xianchao Wang*
[*] *e-mail: zhujq@hit.edu.cn; tianyu_wang@hit.edu.cn; lin_wu@sutd.edu.sg; eleqc@nus.edu.sg*

S1: The mechanical structures and application scenarios for origami metamaterials

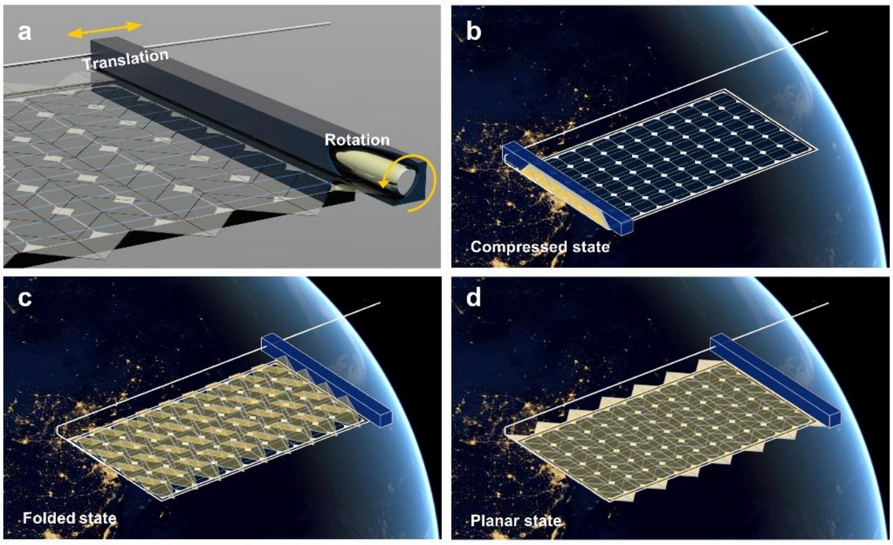

Fig. S1 | Conceptual illustration of origami metamaterial folding process on the solar panel of a satellite. a The diagram of the mechanical structure for origami metamaterial deformation. **b-d** The states of deformation for the proposed origami metamaterial in practical space applications.

As illustrated in Fig. S1, an initial mechanical design with two degrees of freedom is introduced to facilitate the folding of the origami metamaterial above the solar panels. This design enables variations in the folding angle through translation while allowing for rotation, effectively rolling the origami metamaterial into the structure. In this way, the origami metamaterial is in its compressed state before the rocket is launched, reducing the storage space. Once the satellites are deployed in space, the mechanical structure effectively transforms the origami metamaterial from its compressed state to the folded state and planar state covering the whole solar panel. The detailed deformation process can be found in Supplementary Movies 2.

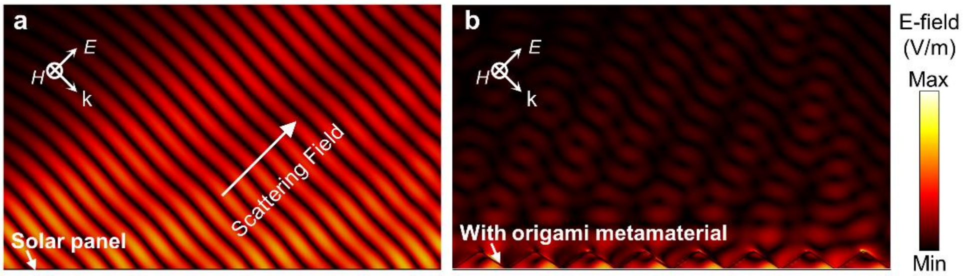

Fig. S2 | The simulated scattering field distribution under 10 GHz planewave oblique incidence. a The solar panel. **b** The solar panel covered by proposed origami metamaterials.

Furthermore, the scattering field distribution of the solar panel with or without origami metamaterial is shown in Fig. S2. As can be seen, the origami metamaterial effectively suppresses the scattering field along the specular direction, which leads to typical applications:

(1) *Improve satellite communications quality*: The origami metamaterial effectively suppresses background noise, hence making communication signals more prominent and thereby improving communication quality.

(2) *Reduce satellite scattering*: The origami metamaterial can effectively reduce wideband scattering field, thereby reducing the interference of scattered signals to highly sensitive radio telescopes.

(3) *Reconfigurable invisibility*: By adjusting the folding angle of origami metamaterial, the satellite can effectively switch between visibility and invisibility state over wideband.

S2: Matrix theory of multilayer structure for reflectance and transmittance calculation

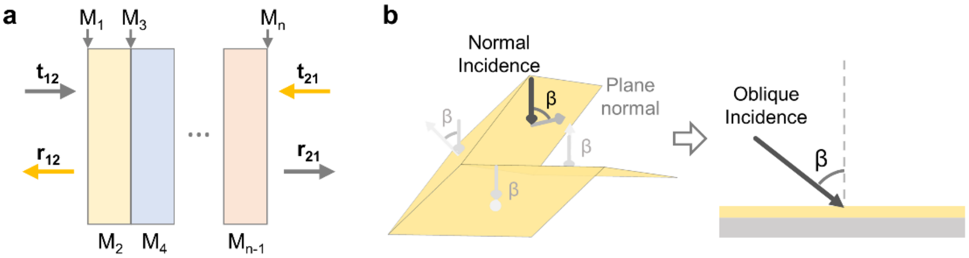

Fig. S3 | The scheme for calculating proposed structure reflectance and transmittance. a The multilayer structure corresponding to the wave-transfer matrices. **b** Equivalent schematic of normal incidence in folded state and oblique incidence in planar state.

Matrix theory is used to calculate the transmittance, reflectance, and absorptance of proposed structure with different fold angle among visible to near-infrared light. The proposed structure is a ITO film on the PET substrate; hence it can be conveniently divided into a concatenation of basic elements described by total wave-transfer matrix M_{total}, whose elements are A, B, C, and D. The multilayer system can be divided into concatenation of basic elements, whose scheme is shown in Fig. S1a.

$$M_{total} = M_N ... M_2 M_1 = \begin{bmatrix} A & B \\ C & D \end{bmatrix} \tag{1}$$

The expression of the wave-transfer matrix of medium and interface at arbitrary incidence angle is shown in details in Saleh's classic book[1]. With the aid of the relation between total wave-transfer matrix M_{total} and scattering matrix S, we can obtain the overall transmittance and reflectance of the multilayer system.

$$S = \begin{bmatrix} t_{12} & r_{21} \\ r_{12} & t_{21} \end{bmatrix} = \frac{1}{D} \begin{bmatrix} AD - BC & B \\ -C & 1 \end{bmatrix} \tag{2}$$

where the t_{12} and r_{12} are the forward amplitude transmittance and reflectance, while t_{21} and r_{21} are the amplitude transmittance and reflectance in the backward direction, respectively. In the planar state, the structure is illuminated by the light perpendicular to the sheet, while in the folded state, the normal of four parallelogram resonators of the unit cell have the same angle β with the incident wave. Hence, the transmittance and reflectance of structure at folded state can be equivalent to the transmittance and reflectance of the structure at planar state under oblique

incidence, as shown in Fig. S1b. Especially, when the folded angle β = 95° of the folded structure, it can be equivalent to the light incident planar structure at an oblique angle of 52.34°.

S3: Simulated sunlight experiment configuration

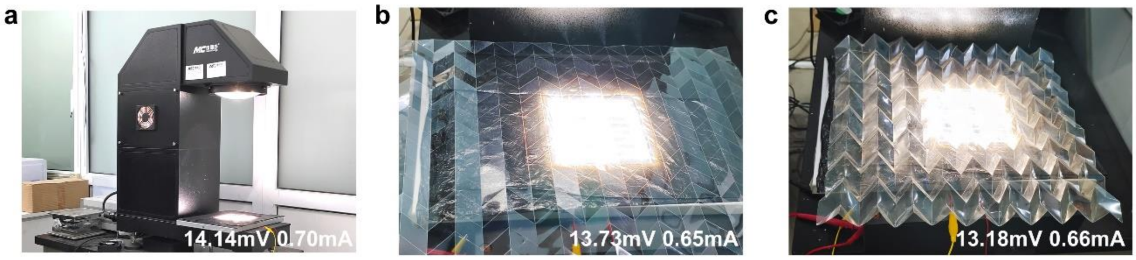

Fig. S4 | The configuration and measured results of simulated sunlight experiment. a The configuration for simulated sunlight with the solar panel placed under the illumination. **b,c** The scene of solar panel under the cover of proposed metamaterial at planar and folded state, respectively. The measured open circuit voltage and short circuit current are shown in the images.

S4: Theory of multipole decomposition

The electromagnetic waves governed by the electric field $\mathbf{E}(x)$ and the magnetic field $\mathbf{H}(x)$ satisfy the following time-harmonic Maxwell equations

$$\begin{aligned}\nabla\times\mathbf{E}-i\omega\mu_0\mathbf{H}=0,\\ \nabla\times\mathbf{H}+i\omega\varepsilon\mathbf{E}=\mathbf{J},\end{aligned} \tag{1}$$

where ε and μ_0 are respectively permittivity and vacuum magnetic permeability. Moreover, $\mathbf{J}(x)$ denotes the electric current density and it can be viewed as the electromagnetic source. By eliminating $\mathbf{H}$ in (1), one can obtain

$$\nabla\times\nabla\times\ \mathbf{E}-k^2\mathbf{E}=i\omega\mu_0\mathbf{J},$$

where $k=\omega\sqrt{\mu_0\varepsilon}$ denotes the wave number. Using the vectorial Green function, then the electric field can be represented by

$$\mathbf{E}(x)=i\omega\mu_0\left(\mathbb{I}+\frac{1}{k^2}\nabla\nabla\cdot\right)\int_{V_s}G(x,\ y)\ \mathbf{J}(y)\mathrm{d}y,$$

where $\mathbb{I}$ is the 3×3 identity matrix and

$$G(x,y)=\frac{e^{ik|x-y|}}{4\pi|x-y|}, \quad x\neq y,$$

is the fundamental solution to the Helmholtz equation $\Delta u+k^2u=0$. It is well known that the radiating solution $\mathbf{E}$ to the Maxwell equations has the following asymptotic form [2]

$$\mathbf{E}(x)=\frac{e^{ik|x|}}{|x|}\left\{\mathbf{E}^{\infty}(x)+\mathcal{O}\left(\frac{1}{|x|}\right)\right\}, \quad |x|\to\infty,$$

where $\mathbf{E}^{\infty}$ is the so-called electric far-field pattern and it is given by [3]

$$\mathbf{E}^{\infty}(x)=\frac{i\omega\mu_0}{4\pi}\left(\mathbb{I}-xx^{\top}\right)\int_{V_s}e^{-ikx\cdot y}\ \mathbf{J}(y)\mathrm{d}y. \tag{2}$$

Next, we shall use the multipole decomposition method to establish the relationship between the far-field and the electric current density. The multipole decomposition can be obtained by the long-wavelength approximation in reference 4 and we briefly describe it in the sequel. Let θ denote the angle between $\hat{x}$ and y, combing equation (2) and the Jacobi-Anger expansion

$$e^{-ikx\cdot y}=\sum_{n=0}^{\infty}(-i)^n(2n+1)j_n(k|y|)P_n(\cos\theta),$$

one can derive that

$$\mathbf{E}^{\infty}(x)=\frac{i\omega\mu_0}{4\pi}\left(\mathbb{I}-xx^{\top}\right)\sum_{n=0}^{\infty}\mathbf{S}_n, \tag{3}$$

where

$$\mathbf{S}_n=(-i)^n(2n+1)\int_{V_s}j_n(k|y|)P_n(\cos\theta)\ \mathbf{J}(y)\mathrm{d}y.$$

Here j_n are spherical Bessel functions of the first kind with order n and P_n are Legendre polynomials. By a direct calculation and reformulating the formula (3), one has

$$\begin{aligned}\mathbf{E}^{\infty}(x)&=\frac{i\omega\mu_0}{4\pi}\left(\mathbb{I}-xx^{\top}\right)(\mathbf{S}_0+\mathbf{S}_1+\mathbf{S}_2+\mathbf{S}_3+\cdots)\\&\simeq\frac{k_0^2}{\varepsilon_0}\Big\{[x\times[\mathbf{p}_0+\mathbf{T}_2]\times x]+\frac{1}{v_d}[\mathbf{m}_1\times x]\\&\quad+\frac{ik}{6}[x\times[x\times(\hat{Q}_1+\hat{Q}_3)x]]+\frac{ik}{2v_d}[x\times\hat{M}_2x]\\&\quad+\frac{k^2}{6}[x\times[x\times\hat{O}_2^{(e)}xx^{\top}]]+\frac{k^2}{6v_d}[x\times\hat{O}_3^{(m)}xx^{\top}]\Big\}\end{aligned} \tag{4}$$

where $k_0=\omega\sqrt{\mu_0\varepsilon_0}$ signifies the wavenumber in vacuum, and k signifies the wavenumber in medium. Here $\boldsymbol{p}_0+\boldsymbol{T}_2$ denotes the electric dipole, m_1 denotes the magnetic dipole, $\hat{Q}_1+$

$\hat{Q}_3$ denotes the electric quadrupole tensor, $\hat{M}_2$ denotes the magnetic quadrupole tensor, $\hat{O}_2^{(e)}$ denotes the electric octupole tensor and $\hat{O}_3^{(m)}$ is the magnetic octupole tensor. For more details, please see reference 5, 6.

We define the radiating power by

$$P = \frac{1}{2}\sqrt{\frac{\varepsilon_0\varepsilon_d}{\mu_0}}\int_{\mathbb{S}^2} |\mathbf{E}^\infty(x)|^2 ds(x). \tag{5}$$

Substituting (4) into (5), thus the radiating power can be divided into different components generated by the multipoles, namely,

$$\begin{aligned} P &= P_{ED} + P_{MD} + P_{EQ} + P_{MQ} + P_{EO} + P_{MO} \\ &= \frac{k_0^4}{12\pi\varepsilon_0^2 v_d \mu_0} |\mathbf{p}_0 + \mathbf{T}_2|^2 + \frac{k_0^4 \varepsilon_d}{12\pi\varepsilon_0^2 v_d} |\mathbf{m}_1|^2 \\ &\quad + \frac{k_0^6 \varepsilon_d}{1440\pi\varepsilon_0^2 v_d \mu_0} \sum_{\alpha\beta} |Q_{1\alpha\beta} + Q_{3\alpha\beta}|^2 + \frac{k_0^6 \varepsilon_d^2}{160\pi\varepsilon_0 v_d} \sum_{\alpha\beta} |M_{2\alpha\beta}|^2 \\ &\quad + \frac{k_0^8 \varepsilon_d^2}{3780\pi\varepsilon_0^2 v_d \mu_0} \sum_{\alpha\beta\gamma} |O_{2\alpha\beta\gamma}^{(e)}|^2 + \frac{k_0^8 \varepsilon_d^3}{3780\pi\varepsilon_0 v_d} \sum_{\alpha\beta\gamma} |O_{3\alpha\beta\gamma}^{(m)}|^2 . \end{aligned}$$

where P_{ED} and P_{MD} represent the power of electric and magnetic dipole, P_{EQ} and P_{MQ} represent the power of electric and magnetic quadrupole, P_{EO} and P_{MO} represent electric and magnetic octupole, respectively. In this work, the current density of the origami metamaterial is approximately solved by commercial software and substituted into the multipole decomposition theory to obtain the power contribution of poles.

S5: The absorption of the Salisbury screen with origami mechanical support

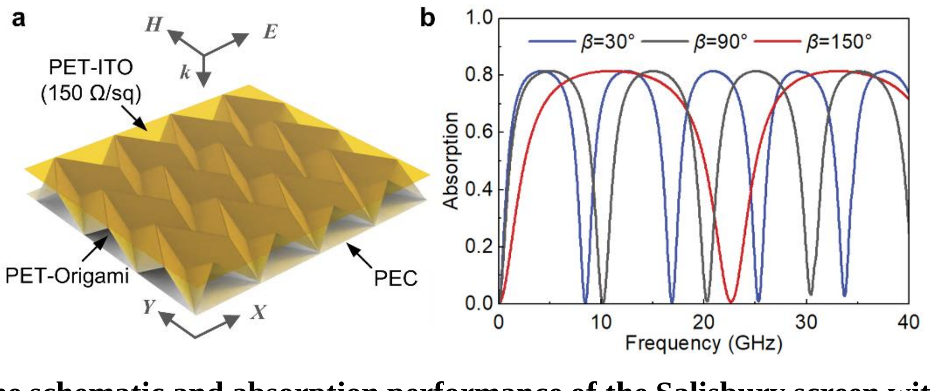

Fig. S5 | The schematic and absorption performance of the Salisbury screen with origami mechanical support. a The schematic of the Salisbury screen using origami structure as

mechanical support with a lossy sheet on the top. **b** The absorption performance of the metamaterial varies with different folded angle *β*.

The Salisbury screen with origami mechanical support is set as a benchmark, whose structure is shown in Fig. S5a. The 0.125 mm-thick PET sheet is used for the origami mechanical support, whose structural parameters are the same as the proposed origami metamaterial. A 0.125 mm-thick PET-ITO sheet with 150Ω/sq surface resistance is placed on the top of the support. By adjusting the folded angle *β* of the origami mechanical support, it becomes possible to effectively control the thickness of the Salisbury screen, whose absorptions are shown in Fig. S5b. As can be seen, the structure achieves intermittent narrowband absorption, with the absorption peak shiftings as the folded angle changes. The narrowband absorption relies on the interference cancellation between the electric ground and the lossy sheet. As a comparison, the proposed origami metamaterial, in its folded state, achieves ultra-wideband continuous absorption due to the interlayer resonance, as illustrated by multipole decomposition.

References

1. Saleh, B. E. A. & Teich, M. C. *Fundamentals of photonics*. (Wiley, 2019).
2. Colton, D. & Kress, R. *Inverse Acoustic and Electromagnetic Scattering Theory*. vol. 93 (Springer New York, 2013).
3. Wang, X., Song, M., Guo, Y., Li, H. & Liu, H. Fourier method for identifying electromagnetic sources with multi-frequency far-field data. *Journal of Computational and Applied Mathematics* **358**, 279–292 (2019).
4. Alaee, R., Rockstuhl, C. & Fernandez-Corbaton, I. An electromagnetic multipole expansion beyond the long-wavelength approximation. *Optics Communications* **407**, 17–21 (2018).
5. Evlyukhin, A. B. & Chichkov, B. N. Multipole decompositions for directional light scattering. *Phys. Rev. B* **100**, 125415 (2019).
6. Evlyukhin, A. B., Fischer, T., Reinhardt, C. & Chichkov, B. N. Optical theorem and multipole scattering of light by arbitrarily shaped nanoparticles. *Phys. Rev. B* **94**, 205434 (2016).
