# Supplementary material for: Origami metamaterials for ultra-wideband and large-depth reflection modulation
Source: Nat Commun. 2024 Apr 12;15:3181. doi: 10.1038/s41467-024-46907-3 (PMC11015009; doi:10.1038/s41467-024-46907-3)
Supplement: Supplementary file 3 — Description of Additional Supplementary Files [file 41467_2024_46907_MOESM3_ESM.docx]

Supplementary Movie 1

“Unfolding process of the fabricated origami metamaterial sample”

Supplementary Movie 2

“Simulated application scenarios of origami metamaterials on satellites”
